# Cash-based assistance and the nutrition status of pregnant and lactating women in the Somalia food crisis: A comparison of two transfer modalities

**DOI:** 10.1371/journal.pone.0230989

**Published:** 2020-04-23

**Authors:** Shannon Doocy, Martin Busingye, Emily Lyles, Elizabeth Colantouni, Bridget Aidam, George Ebulu, Kevin Savage

**Affiliations:** 1 Department of International Health, Johns Hopkins Bloomberg School of Public Health, Baltimore, Maryland, United States of America; 2 Quality Assurance, World Vision Somalia, Mogadishu, Somalia; 3 Department of Biostatistics, Johns Hopkins Bloomberg School of Public Health, Baltimore, Maryland, United States of America; 4 Evidence and Learning Unit, World Vision International, Washington, DC, United States of America; 5 Evidence Building, World Vision International, Geneva, Switzerland; ISDC - International Security and Development Center gGmbH, GERMANY

## Abstract

**Background:**

Large-scale emergency assistance programmes in Somalia use a variety of transfer modalities including in-kind food provision, food vouchers, and cash transfers. Evidence is needed to better understand whether and how such modalities differ in reducing the risk of acute malnutrition in vulnerable groups, such as the 800,000 pregnant and lactating women affected by the 2017/18 food crisis.

**Methods:**

Changes in diet and acute malnutrition status were assessed among pregnant and lactating women receiving similarly sized household transfers over a four-month period (total value of ~US$450 per household) delivered either as food vouchers or as mixed transfers consisting of in-kind food, vouchers, and cash. Baseline and endline comparisons were conducted for 514 women in Wajid, Somalia. Primary study outcomes were Minimum Dietary Diversity for Women, meal frequency, and mid-upper arm circumference (MUAC), with MUAC<21.0 cm classified as acute malnutrition. Adjusted analyses consisted of difference-in-difference analysis using linear and logistic regression models with inverse probability weighting based on propensity scores to account for the non-randomized design.

**Findings:**

No significant difference in change in dietary quality was observed between food voucher and mixed transfer recipients; a significant difference in change in mean meal frequency was observed (0.3 meals/day, CI: 0.1–0.5, p = 0.001) and the mixed transfer group had significantly greater meal frequency at endline (p<0.001). Mean MUAC increased significantly among both voucher (0.9cm, CI: 0.6–1.3, p = 0.001) and mixed transfer recipients (1.3cm, CI: 1.1–1.5, p = 0.001) over the intervention period in adjusted analysis, however, the difference in magnitude of change between the two groups was not statistically significant (0.4cm, CI: -0.1–0.08, p = 0.086).

**Conclusions:**

Within the context of the 2017/18 Somalia food crisis, the modality of assistance provided to pregnant and lactating women (mixed transfers or food-vouchers) made no difference in preventing acute malnutrition and protecting nutritional status.

## Introduction

Somalia has faced nearly three decades of conflict and with a per capita GDP of just US$500, is one of the poorest countries in the world. [1] Despite the establishment of a new government in 2012, conflict persists, and in conjunction with widespread drought in 2016/17, remains a key driver of the continuing food crisis. [2,3] In late 2017, over 3.1 million people in Somalia were facing crisis or emergency levels of food insecurity (Integrated Phase Classification (IPC) Phase 3 and 4) and an additional 3.1 million people were stressed (IPC Phase 2), bringing the total number of people in need of humanitarian assistance at that time to 6.2 million. [4] Included within this population were an estimated 800,000 pregnant and lactating women (PLW) [5] considered a high priority vulnerable group in emergencies due to the increased nutritional demands of pregnancy and lactation who are often targeted for humanitarian assistance.

Despite the record US$27.3 billion allocated to humanitarian responses globally in 2017, the needs of the 201 million people in need of humanitarian assistance outstripped available resources. Somalia was one of seven emergencies in 2018 for which United Nations funding appeals exceeded US$1 billion, but only 68% of the US$1.5 billion request was met. [6] The Grand Bargain, an outcome of the 2016 World Humanitarian Summit, was undertaken to address this persistent humanitarian funding gap, laying out a series of commitments for both donors and implementing agencies to increase efficiency of humanitarian aid. [7] One of the Grand Bargain commitments is to increase the proportion of humanitarian assistance delivered as cash to 25% of all humanitarian assistance by 2020 (as compared to cash accounting for 7% (US$2.0 billion) of international humanitarian assistance in the year preceding the Grand Bargain (2015)). [6,8] Food assistance accounts for the largest form of humanitarian assistance and is a commonly used strategy to maintain and/or improve household food security, thereby preventing acute malnutrition. Food may be financially out of reach more than it is in short supply for most of the population, making cash-based interventions such as cash transfers or vouchers an important option for increasing food access. The choice of transfer modality and whether assistance is delivered in-kind, as food vouchers, or as cash transfers is thus a particularly important issue.

The expansion in the use of cash transfers represents a significant change in the delivery of humanitarian assistance. [9,10] Cash-based approaches are generally perceived as more efficient than in-kind assistance and more supportive of local economies, human agency, and beneficiaries’ dignity. [11] Vouchers are considered a cash-based approach because they are exchanged like cash with some choice, yet differ precisely because that choice is limited (for example to specific foods), which is intended to ensure the outcomes desired by the provider. Vouchers typically have higher implementation costs and because they are less flexible, cannot be directly used by households to meet other priority needs. There is evidence of the positive impact of cash-based approaches on dietary diversity and use of health services from non-crisis contexts, [12] but the link between these and improved nutrition outcomes has not been adequately researched in emergencies. Systematic reviews of cash transfers in humanitarian crises reveal little rigorous evidence as to how cash-based approaches affect nutrition and health outcomes. [13–17] There is limited and sometimes contradictory evidence specifically about the impact of different modalities of assistance or their combinations (e.g. in-kind food provision, vouchers, cash, or mixtures of these modalities) and of programme design and implementation on nutrition status. [14,18] The two reviews that focused on nutrition highlight this dearth of evidence and the need for further research across the spectrum of nutrition in emergencies. [19,20] This includes the application of cash based interventions to the prevention of malnutrition in PLW, which is a common use of both cash and vouchers but of which there have been few studies to determine their comparative effectiveness.

Somalia regularly receives international humanitarian assistance in response to food crises, which is increasingly provided to people through cash or vouchers. In mid-2017 an estimated 3 million people in Somalia were receiving monthly assistance as vouchers or cash, with monthly disbursements totalling US$48 million in May 2017 alone. [21] Our study began in late 2017 when projections indicated the situation would worsen and efforts were underway to increase humanitarian assistance. [22] Sustained humanitarian assistance and above-average rains in the first half of 2018 contributed to a temporary decline in severe food insecurity during the study period. [23] Wajid town, where the study was conducted, is under the control of the national government but besieged by al Shabaab, resulting in access and security concerns, and is also host to a large displaced population from surrounding areas. During the timeframe of the study, food was available in Wajid’s markets; however, food affordability was an issue for many drought-affected households, making cash-based interventions, which are intended to increase access, appropriate.

## Data and methods

The study drew upon two targeted food-assistance interventions with the same objectives that were implemented together in parallel to serve a single overall population of households. The interventions’ targeting criteria were similar but the modalities differed, meaning that the population was divided into two groups: one that received paper food-vouchers, and one that received a combination of in-kind food, electronic vouchers, and unconditional cash (Table 1). This created a natural experiment that allowed the interventions to be compared. The interventions were independent of and pre-dated the development of the study and were outside the control of the researchers, resulting in a non-randomized prospective cohort design for the study. Advanced statistical methods were used to address the limitations of non-random intervention assignment and account for baseline differences in analysis of the two groups receiving assistance. While such a study can have significant limitations, there are few opportunities for rigorous approaches in such highly insecure and acute emergencies where access is severely limited and interventions are designed and implemented in short planning cycles. Taking advantage of such situations is an important way to build a larger humanitarian evidence base and help address evidence gaps such as this one with respect to transfer modality. Whether the choice of modality or spending restrictions (food, cash, or vouchers) can affect outcomes is an important issue for the humanitarian community and particularly in Somalia given the recurrent food crises and prevalence of cash-based assistance.

**Table 1:** Overview of interventions and study participants.

Interventions				
			**Paper Vouchers**	**Mixed Transfers**
**Transfer value**			US$96-130/household/month (transfer value varied monthly)	
**Modalities**			Paper food voucher (US$ 96–130)	In-kind food (US$ 32–45) Food e-voucher (US$ 32–45) Unrestricted cash (US$ 30–50)
**Commodities**			whole grains, flours, pasta, legumes/pulses, vegetable oil	whole grains, flours, pasta, legumes/ pulses, eggs, meat, fruits, vegetables, vegetable oil, milk, sugar, salt, spices
**Total Beneficiary Households (HH)**			1650	3000
**HHs in study communities**^1^			474	700
**HHs in study communities with PLW**^2^			190	280
**Study Participants**				
	**Total**	**Non-Assistance Group**	**FFP Paper Vouchers**	**WFP/UNICEF Mixed Transfers**
**PLW enrolled at baseline**	514	60	166	288
**PLW at endline (% of enrolled)**	490 (95.3%)	59 (98.3%)	162 (97.6%)	269 (93.4%)

^1^Communities of Waberi, Howlwadaag, and El-bon Camp in the District of Wajid

^2^Estimated at 40% of all beneficiary households

**Fig 1:**
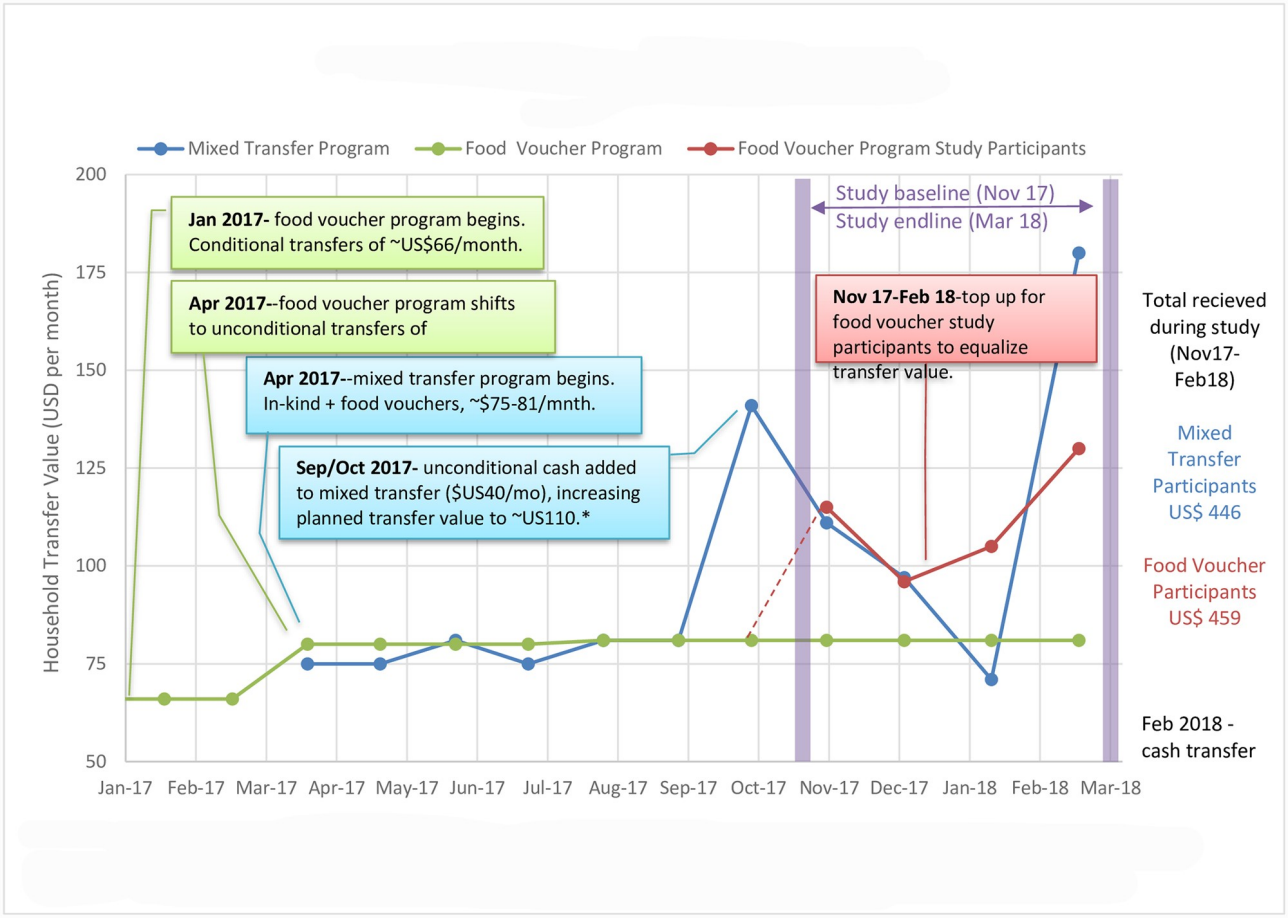
Transfer program evolution over time. In the mixed transfer program, food and in-kind assistance were relatively stable in terms of transfer amount and frequency. Conditional cash was supposed to begin in September, but a bank delay caused the first transfer to be combined with the October transfer. This happened again with the January transfer which was delivered along with the February transfer.

Study locations were selected from a list of intervention neighbourhoods in the town of Wajid based on security and caseload, where locations with more beneficiaries were prioritized. PLW from households meeting vulnerability criteria were invited to be screened, and, if not acutely malnourished (defined as mid-upper arm circumference (MUAC) < 21.0 cm per the national protocol), were invited to enroll in the study. [24] PLW identified as having acute malnutrition were referred to supplementary feeding programmes. PLW households in the mixed transfer study group continued to receive the same transfers as prior to study initiation. To ensure total transfer value during the study period was similar in both intervention groups, PLW households in the food voucher group received a ‘top-up’ voucher in addition to what was received prior to the study (Fig 1).

In addition to intervention recipients, a small number of households not receiving monthly assistance at baseline, but that met vulnerability criteria were also recruited for the study. This “non-assistance” group was recruited from intervention areas and adjacent neighbourhoods and met the same vulnerability criteria as beneficiary households. During the study period, many of these “non-assistance” households also began receiving the interventions or other regular assistance, which limited the value of this comparison group in the final analysis.

Sample-size calculations were conducted using programme data, which indicated that PLW were present in approximately 40% of beneficiary households and acute malnutrition prevalence was 28% among PLW. This translated to an estimated 450 and 135 prospective PLW participants from the mixed transfer and paper-voucher interventions, respectively. Calculations were two-sided and assumed standard deviation for PLW MUAC of 2.5 cm, [25] power = 0.80, and employed a 2:1 ratio for the mixed transfer (n = 260) and paper voucher/non-assistance groups (n = 130) to maximize the ability to detect differences. This sample size was sufficient to detect differences in nutrition outcomes of mean MUAC ≥0.75 cm and food security outcomes ≥10%. A 50% baseline prevalence rate was assumed for food insecurity measures as this is the most conservative estimate; differences of the same magnitude from all other baseline values would be detectable. Diet and acute malnutrition status of children under five years of age were also a primary outcome of this study; however, these findings are presented elsewhere. [26]

Baseline and endline data were collected in November 2018 and March/April 2019 with a four-month intervention period in which transfers were received. Data collection consisted of a 30-minute, questionnaire-based interview focused on socioeconomic status, receipt of assistance, food security, diet, and MUAC as a measure of acute malnutrition status. Primary outcome measures are all commonly used in humanitarian settings and have been validated for use across international settings. These included Minimum Dietary Diversity for Women (MDDW), [27] meal frequency on the preceding day, and MUAC. [24,25] The primary aim of the transfers was to improve household food security, thus Household Hunger Scale (HHS) [28] is also presented to assess the intended outcome of the intervention–household level of food insecurity. MDDW is a summary measure of dietary quality that includes both frequency and diversity of food consumption; women who have consumed at least five of the ten defined food groups on the preceding day are classified as having an adequately diverse diet. The ten food groups included in MDDW are: 1) grains, white roots and tubers, and plantains; 2) pulses; 3) nuts and seeds; 4) dairy; 5) meat, poultry and fish; 6) eggs; 7) dark green leafy vegetables; 8) other vitamin A-rich fruits and vegetables; 9) other vegetables; and 10) other fruits. The number of food groups consumed on the preceding day is reported and MDDW was used together with meal frequency as measures of women’s diets. These were considered intermediate variables that would likely be influenced by regular assistance and reflective of the casual pathway of acute malnutrition resulting from a decline in food intake. MUAC is a commonly used measure for assessment of acute malnutrition status and is more appropriate than body mass index for pregnant women, particularly when gestational age is unknown. Mean MUAC more accurately reflects nutritional status; however, nutrition status is also reported as a dichotomous measure because of its significance in humanitarian response programming where it often determines the type of intervention received.

Interviews were conducted in Somali by female data collectors with prior survey experience who received five days of training on the study, data collection tools, and anthropometric assessment. Focus group discussions were conducted with a subset of participants in both intervention groups at the end of the intervention period to better understand beneficiaries’ experiences and perceptions of transfers. Verbal informed consent was obtained from all women for their household’s participation in the study prior to enrolment and initiation of the first interview; an abbreviated oral consent was used at subsequent data collection points to affirm agreement for continued participation.

Data analysis was performed using Stata 13 (StataCorp, 2013). Descriptive analysis was performed and baseline/endline characteristics were analysed by group. Unadjusted means and prevalence of binary indicators were calculated for each outcome and chi-square and t-test methods were used to assess differences between the intervention groups (vouchers vs mixed transfers) and to compare change from baseline to endline within and between both groups.

At endline, 19% of non-assistance group households reported receiving cash-based assistance in the preceding two months and 30% of PLW reported receiving individual assistance. Due to the small sample size and contamination resulting from other regular assistance received during the study period, the non-assistance group was excluded from statistical hypothesis tests and adjusted regression models using propensity score weighting.

Adjusted analyses used linear models to estimate differences in continuous outcomes between intervention groups from baseline to endline, with main terms for intervention group, time period, and the interaction between intervention group and time period. Logistic models were similarly used to estimate differences in binary outcomes. To account for the non-randomized design, adjusted analyses were conducted using inverse probability of treatment weighting (IPTW) based on household-specific propensity scores. [29,30,31,32,33] Propensity scores were computed using logistic regression to reflect households’ likelihood of receiving the intervention (vouchers vs mixed transfers) given baseline characteristics. Variables predictive of intervention group assignment and used to generate propensity scores included respondent age, education, and marital status; PLW status; household head sex, size, and number of children under five years of age; HHS and meal frequency; and receipt of additional assistance. Propensity scores were used to generate household-specific stabilized weights to adjust for baseline imbalances between intervention groups. [30,33] Distribution of propensity scores and stabilized weights were examined, and covariate balance was evaluated in the unweighted and weighted samples using standardized mean differences between intervention groups. [34] Standardized differences < 0.1 were presumed to indicate a “negligible” difference between groups (see S1 Table). [35] In adjusted data using stabilized weights, standardized differences for all individual and household characteristics with the exception of meals consumed on the preceding day were negligible. The magnitude of the difference in mean meals consumed on the preceding day in unadjusted data was small and considered unlikely to be of importance.

Household-specific stabilized weights were used in linear and logistic models to analyse adjusted change from baseline to endline, as well as differences in change between voucher and mixed transfer beneficiaries. Models utilized cluster-robust standard errors with clustering defined at the household level, allowing for correlation between observations for each woman/household. Coefficients for the interaction of intervention group and time period represent the estimated difference in change comparing the mixed transfer to food voucher beneficiaries.

The study was approved by the Somalia Ministry of Health and the Ministry of Planning and International Cooperation and reviewed by the Institutional Review Board at Johns Hopkins Bloomberg School of Public Health.

## Results

### Study population characteristics

A total of 514 PLW were enrolled in the study, including 288 (56.0%) that received mixed transfers, 160 (31.1%) that received food vouchers, and 60 (11.6%) that did not receive household-level assistance; 95.3% (n = 490) completed the study. Baseline characteristics by group are presented in Table 2. Intervention households were similar with respect to composition; however, women in the voucher group were significantly older and more likely to be pregnant. Food security differed across groups at baseline, which is likely a result of receipt of different assistance packages prior to enrolment as participants all met similar vulnerability criteria. Fewer households in the mixed transfer group had moderate or severe hunger (35.4% compared to 44.0% and 94.9% in voucher and non-assistance groups, respectively) and consumed more meals on the preceding day (2.7 compared to 2.6 and 2.2 in voucher and non-assistance groups, respectively). Most households in the mixed transfer (85.4%) and voucher (62.7%) groups reported individual members receiving other forms of additional assistance as compared to only 25.0% in the non-assistance group.

**Table 2:** Beneficiary and household characteristics at baseline.

		Non-Assistance Group*		Food Voucher Group		Mixed Transfer Group		Assistance groups comparison *P*-value ^1^
		(N = 60)		(N = 166)		(N = 288)		
		Point	95% CI	Point	95% CI	Point	95% CI	
***Respondent Characteristics***								
**Age**	Mean	25.7	(24.7,26.8)	28.6	(27.8,29.4)	27.3	(26.7,27.9)	**0.013**
**Education Level** ^2^								
Never attended		94.9%	(85.9,98.9%)	81.9%	(75.2,87.5%)	88.9%	(84.7,92.3%)	0.091
Some primary but not complete		3.4%	(0.4,11.7%)	13.3%	(8.5,19.4%)	7.3%	(4.6,10.9%)	
Completed primary		1.7%	(0.0,9.1%)	4.8%	(2.1,9.3%)	3.8%	(1.9,6.7%)	
**Marital Status**								
Married monogamous		76.7%	(64.0,86.6%)	62.7%	(54.8,70.0%)	60.8%	(54.9,66.4%)	0.055
Married polygamous		23.3%	(13.4,36.0%)	33.7%	(26.6,41.5%)	38.5%	(32.9,44.4%)	
Widowed, Divorced, Separated		0.0%	(0.0,6.0%)	3.6%	(1.3,7.7%)	0.7%	(0.1,2.5%)	
**Beneficiary Type**								
Pregnant		46.7%	(33.7,60.0%)	50.6%	(42.7,58.4%)	38.2%	(32.6,44.1%)	**0.041**
Lactating		50.0%	(36.8,63.2%)	46.4%	(38.6,54.3%)	59.4%	(53.5,65.1%)	
Pregnant and Lactating		3.3%	(0.4,11.5%)	1.8%	(0.4,5.2%)	2.1%	(0.8,4.5%)	
***Household Characteristics***								
**Female Headed Households**		1.7%	(0.0,8.9%)	4.8%	(2.1,9.3%)	1.7%	(0.6,4.0%)	0.058
**Household Size**	Mean	6.5	(5.8,7.1)	6.6	(6.2,7.0)	6.3	(6.0,6.6)	0.192
**Children < 5 years in HH**	Mean	1.2	(1.0,1.4)	1.4	(1.2,1.5)	1.3	(1.2,1.3)	0.136
% of HH with children < 5 years		81.7%	(69.6,90.5%)	89.2%	(83.4,93.4%)	88.2%	(83.9,91.7%)	0.757
**Household Hunger Scale** ^3^	Mean	2.8	(2.5,3.1)	1.4	(1.2,1.5)	1.1	(0.9,1.2)	**0.007**
Little to no hunger in the household		5.1%	(1.1,14.1%)	56.0%	(48.1,63.7%)	64.6%	(58.8,70.1%)	0.085
Moderate hunger in the household		78.0%	(65.3,87.7%)	41.6%	(34.0,49.5%)	34.7%	(29.2,40.5%)	
Severe hunger in the household		16.9%	(8.4,29.0%)	2.4%	(0.7,6.1%)	0.7%	(0.1,2.5%)	
**Meals consumed on preceding day**	Mean	2.2	(2.1,2.3)	2.6	(2.5,2.7)	2.7	(2.7,2.8)	**0.026**
% consuming one meal or less		0.0%	--	0.0%	--	0.0%	--	---
***Receipt of Food Assistance***								
**Last time household food assistance received**								
Less than one month ago		0.0%	(0.0,6.0%)	100.0%	(97.8,100.0%)	100.0%	(98.7,100.0%)	---
Between 1–2 months ago		0.0%	--	0.0%	--	0.0%	--	
More than 2 months ago		1.7%	(0.0,8.9%)	0.0%	--	0.0%	--	
Don't know		98.3%	(91.1,100.0%)	0.0%	--	0.0%	--	
**Value of assistance (past month; USD)**	Mean	--	--	81.1	(81.0,81.1)	85.0	(85.0,85.0)	***< 0*.*001***
**Additional individual food assistance received** ^4^		25.0%	(14.7,37.9%)	62.7%	(54.8,70.0%)	85.4%	(80.8,89.3%)	***< 0*.*001***
Pregnant woman		1.7%	(0.0,8.9%)	25.9%	(19.4,33.3%)	20.5%	(16.0,25.6%)	0.183
Lactating woman		1.7%	(0.0,8.9%)	21.1%	(15.1,28.1%)	10.4%	(7.1,14.5%)	**0.002**
Child <5 years, not malnourished		10.0%	(3.8–20.5%)	20.5%	(14.6–27.4%)	57.6%	(51.7–63.4%)	***< 0*.*001***
Malnourished Child		11.7%	(4.8–22.6%)	7.2%	(3.8–12.3%)	9.4%	(6.3–13.3%)	0.432
School feeding		1.7%	(0.0,8.9%)	9.0%	(5.1,14.5%)	35.8%	(30.2,41.6%)	***< 0*.*001***

*Household food assistance was not received at enrollment; however, many households in this group began receiving assistance during the study period

^1^Two intervention group comparison using Pearson's chi-square for proportions and t-test for means

^2^No women completed secondary schooling

^3^HHS is six-point scale depicting hunger in the past month; 0–1 is classified as little/no hunger, 2–3 as moderate hunger, and 4–6 as severe hunger

^4^Each assistance type as a % of all HHs; some households received multiple types of individual assistance

### Household food security

**Fig 2:**
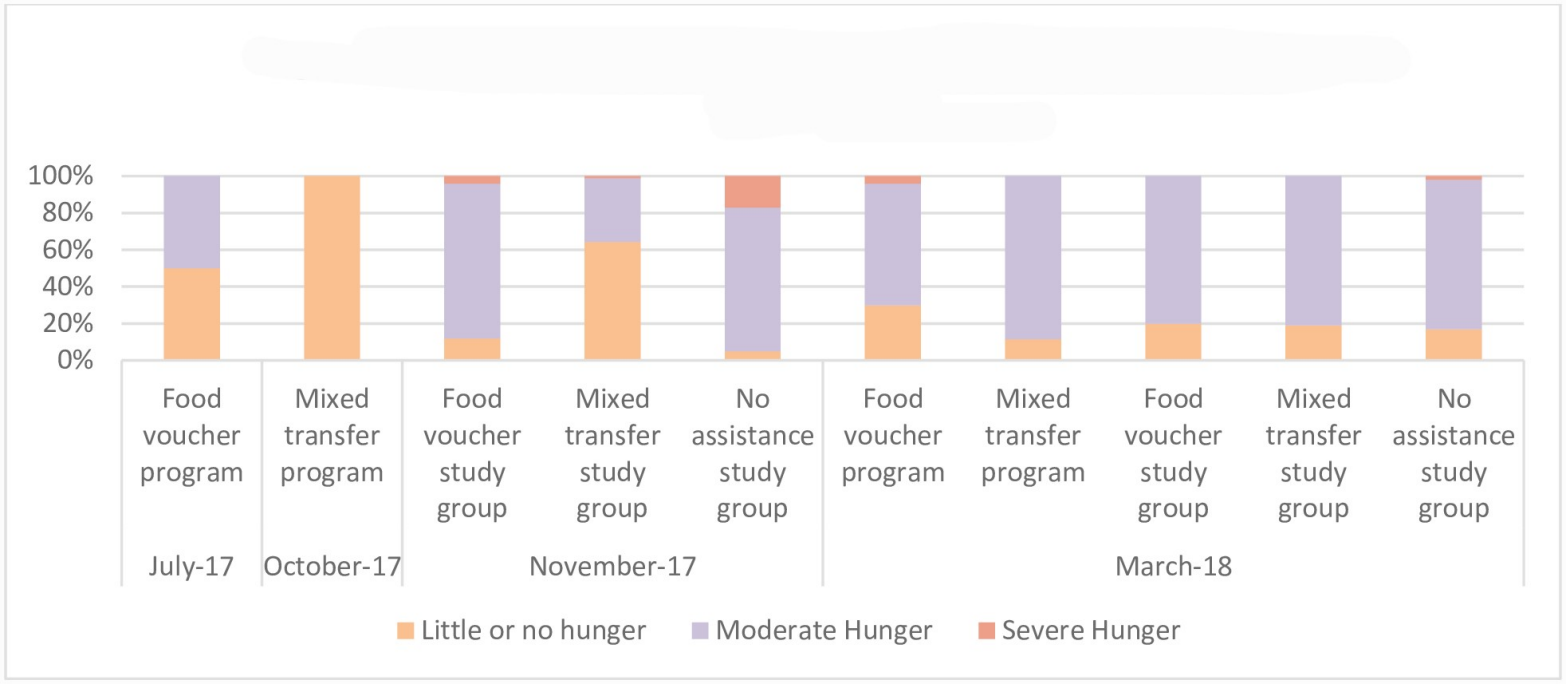
Household food security among beneficiary and study households.

Food security, measured using the HHS, is presented for the interventions’ beneficiary populations (drawing on the interventions’ regular post-distribution monitoring (PDM)) and for the study population in Fig 2. While PDM data was not available at the same time point for voucher and mixed transfer beneficiaries prior to the study, at the time of the study initiation both the mixed transfer and voucher study groups were more food-secure than the non-assistance study group. In focus groups, intervention recipients reported that transfers were the primary household food source and comprised most of the households’ income; income generation opportunities were limited and primarily included casual labour and selling goods in markets.

Household food security measures at endline and change from baseline to endline for the study population are presented in Table 3. At endline, the non-assistance group had the lowest meal frequency with an average of 2.0 meals daily and 10.2% consuming one meal or less per day. Meal frequency at endline was significantly greater among mixed transfer recipients as compared to voucher recipients (2.7 vs 2.4 mean meals per day, p<0.001; 8.0% vs 2.2% consuming one meal or less per day, p = 0.005). At endline, food security, as measured by HHS, was similar between the intervention groups with a mean HHS score of 1.8 and approximately 80% of households having moderate hunger and 19% no hunger. Only the non-assistance group had severely food insecure households (1.7%). Overall, household food security indicators declined over the study period in both intervention groups. When change over time was assessed, the non-assistance group was the only group to see improvements in HHS score (likely due to lower baseline values and a large proportion of households in this group gaining access to expanded humanitarian assistance programmes). The increase in HHS score compared across the two intervention groups was significantly greater among mixed transfer households as compared to vouchers. There was no significant change in mean meal frequency in any of the groups over the intervention period.

**Table 3:** Endline differences and change over time in household food security.

		Non-Assistance Group*		Food Voucher Group		Mixed Transfer Group		Assistance groups comparison *P*-value ^1^
		(N = 59)		(N = 162)		(N = 269)		
		Point	95% CI	Point	95% CI	Point	95% CI	
***Characteristics at Endline***								
**Household Hunger Scale** ^2^	Mean	1.9	(1.7,2.1)	1.8	(1.7,1.869)	1.8	(1.8,1.9)	0.403
Little to no hunger in the household		16.9%	(8.4,29.0%)	19.8%	(13.9,26.7%)	19.3%	(14.8,24.6%)	0.915
Moderate hunger in the household		81.4%	(69.1,90.3%)	80.2%	(73.3,86.1%)	80.7%	(75.4,85.2%)	
Severe hunger in the household		1.7%	(0.0,9.1%)	0.0%	(0.0,2.3%)	0.0%	(0.0,1.4%)	
**Meals consumed on preceding day**	Mean	2.0	(1.9,2.2)	2.4	(2.3,2.6)	2.7	(2.6,2.7)	***< 0*.*001***
% consuming one meal or less		10.2%	(3.8,20.8%)	8.0%	(4.3,13.3%)	2.2%	(0.8,4.8%)	**0.005**
***Change from Baseline to Endline***								
**Household Hunger Scale** ^2^	Mean	-0.9	(-1.3,-0.5)	0.4	(0.2,0.6)	0.8	(0.6,0.9)	**0.004**
Little to no hunger in the household		11.9%	(0.8,23.0%)	-36.3%	(-46.0,-26.5%)	-45.3%	(-52.5,-38.0%)	0.252
Moderate hunger in the household		3.4%	(-11.1,17.9%)	38.7%	(29.0,48.4%)	45.9%	(38.7,53.2%)	
Severe hunger in the household		-15.3%	(-25.4,-5.1%)	-2.4%	(-4.7,-0.1%)	-0.7%	(-1.7,0.3%)	
**Meals consumed on preceding day**	Mean	-0.2	(-0.4,0.0)	-0.2	(-0.3,0.0)	-0.1	(-0.2,0.0)	0.248
% consuming one meal or less		10.2%	(3.8,20.8%)	8.0%	(4.3,13.3%)	2.2%	(0.8,4.8%)	**0.005**

*Household food assistance was not received at enrollment; however, many households in this group began receiving assistance during the study period

^1^Two intervention group comparisons using Pearson's chi-square for proportions and t-test for means

^2^Household Hunger Scale is six-point scale of hunger in the past month (0–1 is classified as little/no hunger, 2–3 as moderate hunger, and 4–6 as severe hunger)

^3^Because baseline is 0% in all groups, comparison of change is equivalent to endline values

### Women’s dietary diversity and meal frequency

Primary study outcomes concerning PLW diet and MUAC are summarized in Table 4. Meal frequency was constant throughout the study period for PLW in all three groups with both intervention groups consuming an average of 2.5 meals per day compared to 2.0 meals in the non-assistance group. While meal frequency was similar among PLW in both intervention groups at baseline, it was significantly greater in the mixed transfer group at endline (2.6 vs 2.4 meals daily, p<0.001). In adjusted models, a modest yet significant decrease in PLW meal frequency was observed among voucher recipients, whereas a modest, also significant, increase was found for mixed transfer recipients (difference in baseline/endline change between groups of 0.3 meals daily, p = 0.001).

**Table 4:** Group differences and change over time in pregnant and lactating women dietary and nutrition outcomes.

	Non-Assistance Group*		Food Voucher Group		Mixed Transfer Group		Assistance groups comparison *P*-value ^1^
	(N = 60/59)		(N = 166/162)		(N = 288/269)		
	Point	95% CI	Point	95% CI	Point	95% CI	
**DIETARY OUTCOME MEASURES**							
***Meals Consumed on the Preceding Day (mean)***							
Baseline	2.0	(1.84,2.13)	2.5	(2.4,2.5)	2.5	(2.4,2.5)	0.834
Endline	2.0	(1.82,2.15)	2.4	(2.3,2.5)	2.6	(2.5,2.6)	**0.009**
Baseline/Endline Change (unadjusted)	0.00	(-0.2,0.2)	-0.04	(-0.2,0.1)	0.1	(0.0,0.2)	0.086
Baseline/Endline Change (adjusted) ^2^			-0.2	(-0.3,0.0)	0.1	(0.0,0.2)	**0.001**
Difference intervention groups (adjusted) ^2^			**0.3 (0.1,0.5) p = 0.001**				
***Food Groups Consumed on the Preceding Day (mean)***							
Baseline	4.0	(3.5,4.4)	4.8	(4.5,5.1)	5.3	(5.1,5.4)	**0.008**
Endline	4.9	(4.5,5.3)	5.2	(4.9,5.4)	6.0	(5.8,6.2)	***< 0*.*001***
Baseline/Endline Change (unadjusted)	0.9	(0.4,1.5)	0.4	(0.0,0.8)	0.7	(0.5,1.0)	0.118
Baseline/Endline Change (adjusted) ^2^			0.5	(0.0,0.9)	0.7	(0.5,1.0)	0.324
Difference between intervention groups (adjusted) ^2^			0.3 (-0.3,0.8) p = 0.324				
***Percent Achieving Minimum Dietary Diversity for Women (MDDW)***							
Baseline	51.7%	(38.4,64.8%)	63.3%	(55.4,70.6%)	72.9%	(67.4,78.0%)	**0.031**
Endline	72.9%	(59.7,83.6%)	67.3%	(59.5,74.4%)	86.6%	(82.0,90.4%)	***< 0*.*001***
Baseline/Endline Change (unadjusted)	21.2%	(7.3,35.1%)	4.0%	(-6.3,14.3%)	13.7%	(7.3,20.1%)	**0.028**
Baseline/Endline Change (adjusted) ^2^			6.2%	(-7.0,19.4%)	13.9%	(7.4,20.5%)	0.303
Difference between intervention groups (adjusted) ^2^			7.7% (-7.0%,22.5%) p = 0.303				
**NUTRITION OUTCOME MEASURES**							
***Mid-Upper Arm Circumference (mean)***							
Baseline	25.2	(24.8,25.6)	24.4	(24.2,24.7)	25.2	(25.0,25.5)	***< 0*.*001***
Endline	25.6	(24.9,26.3)	25.4	(25.0,25.7)	26.5	(26.3,26.8)	***< 0*.*001***
Baseline/Endline Change (unadjusted)	0.4	(-0.3,1.2)	0.9	(0.6,1.3)	1.3	(1.1,1.5)	0.065
Baseline/Endline Change (adjusted) ^2^			0.9	(0.6,1.3)	1.3	(1.1,1.5)	0.086
Difference between intervention groups (adjusted) ^2^			0.4 (-0.1,0.8) p = 0.086				
***Acute Malnutrition Prevalence (based on national threshold*, *MUAC<21*.*0cm)***							
Baseline	0.0%	--	0.0%	--	0.0%	--	---
Endline	5.1%	(1.1,14.1%)	3.1%	(1.0,7.1%)	0.0%	(0.0,1.4%)	**0.004**
Baseline/Endline Change (unadjusted)	5.1%	(-0.5,10.7%)	3.1%	(1.0,7.1%)	0.0%	(0.0,1.4%)	**0.004**
Baseline/Endline Change (adjusted) ^2^			2.9%	(-0.4,6.1%)	0.0%	—	0.086
Difference between intervention groups (adjusted) ^2^			-2.9% (-6.1,0.4%) p = 0.086				

*Household food assistance was not received at enrollment; however, many households in this group began receiving assistance during the study period

^1^Baseline and endline two intervention group comparison using Pearson's chi-square for proportions and t-test for means

^2^Adjusted analyses included inverse probability weighting (to account for the non-randomized design)

Dietary diversity, or the number of food groups consumed on the preceding day and those achieving MDDW, differed significantly at baseline and endline between groups and was greatest among PLW receiving mixed transfers, followed by food vouchers, at both time periods. The non-assistance group consistently had the lowest dietary diversity. An increase in the number of food groups consumed on the preceding day (between 0.4 to 0.9 food groups per day) was seen in all three groups, though increases were only statistically significant in the mixed transfer and non-assistance groups. In adjusted analysis, increases of 0.5 (CI: 0.0–0.9) and 0.7 (CI: 0.5–1.0) food groups per day were observed among PLW in the voucher and mixed transfer groups, respectively; however, there was no significant difference in the magnitude of improvement between groups in adjusted models (0.3, p = 0.324).

The mixed transfer group, followed by the voucher group, had the largest proportion of PLW achieving MDDW at baseline and endline. The differences observed between the intervention groups at both baseline and endline were significant. In unadjusted analysis, the non-assistance group saw the largest improvement in MDDW at 21.2% (from 51.7% to 72.9%), followed by the mixed transfer group (13.7% improvement, from 72.9% to 86.6%) and the voucher group (4.0% improvement, from 63.3% to 67.3%). In adjusted models, there was no significant difference in the proportion of PLW achieving MDDW between the mixed transfer and voucher group (7.7%, CI: -7.0–22.5%, p = .303). In focus group discussions, intervention recipients reported that PLW and young children were prioritized to receive more and better food than other household members; increases in dietary diversity, meal frequency, and consumption of high-quality foods were attributed to transfer receipt. It was also reported that women were the primary decision-makers in food allocation and use of transfers, or that these decisions were made jointly. In the mixed transfer group, concerns about appropriate use of cash were not reported and overall, mixed transfers were preferred because they were more flexible and allowed households to meet a greater variety of needs.

### Women’s mid-upper arm circumference

PLW mean MUAC at baseline was 25.2 cm in the non-assistance and mixed transfer groups but lower (24.4 cm) in the food voucher group. At endline, mean MUAC remained significantly greater in the mixed transfer group (26.5 cm vs 25.4 cm, p<0.001). In unadjusted analysis, mean MUAC increased during the study period for all intervention groups with the smallest increase observed in the non-assistance group (0.4 cm, CI: -0.3–1.2) and greater improvements in the food voucher (0.9 cm, CI: 0.6–1.3) and mixed transfer (1.3 cm, CI: 1.1–1.5) groups. In adjusted analysis, mean MUAC increased by an average of 0.4 cm (CI: -0.1–0.8, p = 0.086) more in the mixed transfer group compared to the voucher group, though the observed difference was not statistically significant.

No PLW receiving mixed transfers developed acute malnutrition during the study period. In contrast, 5.1% (CI: 0.0–10.7) of non-assistance PLW and 3.1% (CI: 0.0–6.1) of PLW receiving food vouchers were classified as having acute malnutrition at endline. In adjusted models, the increase in acute malnutrition prevalence from baseline to endline was 2.9% (CI: -0.4–6.1%) greater among voucher beneficiaries as compared to mixed transfer beneficiaries, a difference that was not statistically significant (p = 0.086).

## Discussion

When the study was conducted in 2017, 17% of international humanitarian assistance programmes in Somalia had a cash transfer element, including 11% that were wholly cash and 6% that included cash among other modalities. [6] Most cash assistance was unconditional (92%) and unrestricted (57%), and delivered as e-vouchers (35%) or mobile money (32%). [21] A review of the joint performance and impact of cash-based assistance (cash or vouchers) found that cash-based assistance is now routine in Somalia and that the 2017 response was perceived as more effective than prior responses. It found no significant issues related to the provision of household assistance predominantly to women and a key recommendation of the review was to reconsider whether restrictions (i.e., vouchers) are in fact needed: vouchers may not be necessary in locations where markets can provide appropriate items and cash provides greater flexibility, is preferred by beneficiaries, and its use is increasingly encouraged by donors. [36]

At study initiation in November 2017, Wajid was classified as facing a food emergency (IPC level 4). Deyr rains occurring from October to December were below average, prompting projections of decreased crop yields, risk of famine, and calls to scale up humanitarian assistance in Southern Somalia. [23] By the end of the study period, post-harvest seasonal improvements in food and income sources as well as humanitarian assistance had contributed to improvements in regional food security such that the risk of famine was declining despite projections of food and income sources remaining below average through mid-2018. [37] At endline, food security was similar between the intervention groups in our study, with approximately 80% of households experiencing moderate hunger and 19% little or no hunger. Only the non-assistance group had any severely food-insecure households (1.7%). For the intervention groups, an overall decline in household food security was observed during the study period. The non-assistance group saw an improvement but this was likely due to their initial situation being much worse and, crucially, to many of them gaining access to humanitarian assistance (18.6% gained access to household assistance and 25–30% of PLW and children under five years of age were receiving individual assistance at endline). Humanitarian assistance in any form was playing a critical role in buffering households against food insecurity, even if not sufficient to fully secure them.

Despite the overall decline in household food security, individual outcomes for PLW were more encouraging. With respect to diet, the mixed transfer group saw a slight improvement in frequency of meal consumption and dietary diversity was greatest among the mixed transfer group at endline. This group also saw the largest increase in the proportion of women achieving minimum dietary diversity. Gains observed in the mixed transfer group were greater but statistically similar to those in the food voucher group, indicating that mixed transfers were at least as effective as food vouchers and suggesting that unconditional cash transfers, which are typically less costly to deliver than vouchers [11], may perform equally well in the Somalia context. While relatively few other studies report specifically on maternal outcomes of cash transfers in food insecure settings, our findings are consistent with impacts reported elsewhere. In Somalia, a study comparing cash transfers to no assistance in Mogadishu IDP camps found that PLW who received cash had improved dietary diversity; in Pakistan, a study of women receiving both cash transfers and fresh food vouchers saw gains in dietary diversity, though the magnitude of improvement was greater among mothers receiving cash. [38]

The changes in MUAC experienced by PLW receiving mixed transfers and those receiving vouchers would be expected to have meaningful impact on maternal and child outcomes such as improving infant survival and reducing low birth weights, intrauterine growth retardation, and pre-term birth. [39] The observed gains are likely attributable to women’s high level of control of household food resources and relatively prominent male support for prioritization of PLW and young children when food is limited observed in our qualitative data.

Household-level assistance, delivered either as mixed transfers or vouchers, has individual-level benefits for PLW within them; this is an important finding for Somalia and emergency assistance programmes more generally because it is more easily delivered at scale. Targeting of households with PLW could be an effective and more efficient of means of improving maternal diet and nutrition than both individual PLW and household level transfers delivered in parallel. Few previous studies have reported maternal nutrition status as an outcome of cash transfer programmes and additional studies in other contexts are needed. Our findings align with other recent research in Somalia that reported a mean increase of 1.1 cm in maternal MUAC among cash transfer recipients in Mogadishu IDP camps; the study in Pakistan observed no gains in maternal MUAC among transfer recipients despite improvements in dietary diversity. [39] In our study, very few women developed acute malnutrition during the study period and only the mixed transfer group had no women acutely malnourished at endline. When interpreting these positive findings with respect to MUAC and prevalence of acute malnutrition, it is critical to consider that other individually targeted interventions for PLW were scaled up during the study period, thus the observed gains in maternal MUAC cannot be attributed to the study interventions alone.

Findings from this study align with the 2017 Somalia cash review and with other research from Somalia specific to PLW in not observing a difference between mixed transfers and food vouchers in the impact on the nutrition status of pregnant and vulnerable women.

## Limitations

There are considerable challenges to conducting rigorous research in humanitarian settings; however, the value of impact evaluations can be substantial given the paucity of available evidence to inform humanitarian programming. [40] This study was no exception and had the following principal limitations associated with implementation in acute crisis setting: 1) inability to randomize due to ongoing assistance programming; 2) shorter than anticipated study period (4 months vs 6 months) due to lack of programme continuity; 3) challenges in identifying and maintaining a non-assistance comparison group due to the high availability of assistance and imperative to assist to all in need; and 4) seasonality and regional improvements in food security, which also likely influenced the outcomes of study.

The small non-assistance comparison group and abbreviated study period both likely contributed to a reduced ability to detect statistically significant differences for many outcomes. Another potential concern is that households reported in focus groups that each monthly transfer lasted between 10–20 days, making the timing of measurements with respect to the last transfer potentially important. Efforts were made to plan data collection across the groups at similar time points relative to the transfers, but it is possible that timing inconsistencies may have influenced outcome measures differentially across groups (where food security and diet indicators may be expected to decline as time since transfer receipt increases). Finally, inconsistencies in transfer timing and amount occurred despite the total transfer over the study period being similar, which may have influenced behaviour of transfer recipients and affected outcome measures. Future research of food security and nutrition in crisis contexts where monthly food or cash assistance is prevalent and actors and targeting practices are likely to vary over time should consider increased sample size where feasible as a means of improving statistical power and the ability to significantly detect more modest differences.

## Conclusions

In 2017/18, Somalia faced its fifth consecutive year of below average rainfall and populations in Wajid District and elsewhere were also impacted by conflict and displacement. [38] Provision of in-kind food, food vouchers, and cash transfers are all common mechanisms for providing assistance in humanitarian emergencies. This is particularly true in Somalia, where cash accounted for 17% of international humanitarian assistance in 2017. An estimated US$2.8 billion of humanitarian assistance was provided in the form of cash and vouchers in 2016, and in 2017 US$1.2 billion in cash assistance was reported through the financial tracking service. [6] Vouchers and cash transfers may be superior to in-kind assistance for improving dietary diversity (albeit not caloric intake) [41] but other logistical and contextual considerations typically drive decisions on how humanitarian assistance is provided. The majority of cash assistance provided is at the household level and evaluations rarely quantify individual level impacts despite concerns that the benefits of assistance may vary based on intra-household decision making and resource allocation, which may differ by transfer modality. PLW recipients of vouchers and mixed transfers had similar outcomes with respect to acute malnutrition status. Both groups also had similarly large increases in mean MUAC over the four-month study period. Our findings are similar to other studies of cash assistance in Somalia in indicating that mixed transfers, inclusive of unconditional cash, are equally or more beneficial for PLW than food vouchers. Given beneficiary preferences for cash, potential lower costs of cash compared to vouchers, and positive nutritional outcomes among PLW achieved with cash, there is a strong body of evidence to support the use of cash transfers for protecting maternal nutrition in Somalia.

## Supporting information

S1 Table Baseline characteristics by assistance group in unadjusted data and adjusted using stabilized inverse probability weighting*. (DOCX)

S1 File (PDF)
