# Supplementary material for: Cash-based assistance and the nutrition status of pregnant and lactating women in the Somalia food crisis: A comparison of two transfer modalities
Source: PLoS One. 2020 Apr 23;15(4):e0230989. doi: 10.1371/journal.pone.0230989 (PMC7179869; doi:10.1371/journal.pone.0230989)
Supplement: S1 File — (PDF) [file pone.0230989.s002.pdf]

CASH AND VOUCHERS FOR NUTRITION IN WAJID SOMALIA

Research for Health in Humanitarian Crises (R2HC)

Food & Nutrition Crises Call 2017

Lead research partners	World Vision and Johns Hopkins School of Public Health
Project Length	18 months
Location	Wajid District
Investigators	Shannon Doocy, Kevin Savage, Bridget Aidam, George Ebulu, Martin Busingye

Purpose:

The aim of the project is actionable recommendations to inform future food and nutrition programming in Somalia and other food crises. It focuses on prevention of acute malnutrition among vulnerable populations, including pregnant and lactating women and children under five years of age.

There is limited and sometimes confusing evidence about the impact of combinations of assistance modalities, such as cash transfers and food vouchers, on nutritional status and about the importance of the design and implementation of such programmes. We intend to compare the effectiveness of food vouchers to the effectiveness of mixed transfers, consisting of food vouchers and unconditional cash transfers: is the provision of both cash and vouchers more effective than vouchers alone for preventing acute malnutrition and supporting health behaviours and food security among vulnerable groups (pregnant and lactating women and children under five years of age).

Expected outcomes:

This research will provide much needed evidence on the impact of modalities on nutrition and health outcomes for pregnant and lactating women and children under five years of age, and make an important contribution to the expansion of cash programming in future humanitarian crises. Findings will be directly utilized to improve health and nutrition status in the current famine response, through the incorporation of implications, learning and identified successful innovations into refinement of future programs implemented by World Vision and partners, by applying the evidence to support the scaling up of successful interventions and practices to other World Vision programmes in humanitarian contexts, and we anticipate that by publishing and sharing findings, lessons learned and good practices with key stakeholders and strategic partners these will be applied in Somalia and other similar contexts. At least two articles will be submitted for publication in an open-access journal, and briefing sheets and presentations will be prepared to summarize key findings for dissemination events.

Research Question

Are household transfers comprised of food, food voucher and cash more effective than food vouchers alone, in preventing malnutrition and supporting health behaviour among pregnant and lactating women?

Design and Methods

A quasi-experimental three arm parallel study of the impact of different modalities of emergency food and nutrition interventions on the nutrition outcomes of pregnant and lactating women, and the under-5 children in their households.

Using mixed-methods we will quantitatively measure nutrition outcomes and qualitatively investigate how household members utilize and perceive the different types of assistance, its objectives and implementation.

Setting

The research will be conducted in Wajid District, Bakool Region, Somalia between July 2017 and April 2018.

Research will be within the emergency food security, health and nutrition programming implemented by World Vision on behalf of WFP, FFP and UNICEF which consists of two parallel projects with the same intended outcomes. The introduction of a new complementary component (cash transfers) to one of them, provides a unique opportunity to employ a rigorous methodological approach for comparison of the combined interventions; which are 1) food and food-vouchers—WFP monthly e-cards flexibly redeemable at any time, frequency, or amount, combined with in-kind food, approximately $81US total value (3000 households); 2) food-vouchers only—paper, distributed and redeemed in full monthly of approximately $81 US value (1450 households); and 3) cash–$ 35US/month provided additionally to the WFP food and food voucher intervention from mid-2017. The food vouchers have similar targeting/vulnerability criteria and are provided primarily to women; the cash is without conditions, labelled a 'top-up' to the food vouchers for other, non-food needs.

Approvals

1. **Ministry of Health**

   Name & Title........Isaak Mohamud Mursal Director General

   Signature........[signature]

   Date........1-11-2017

2. **Ministry of Planning & International Cooperation**

   Name & Title........Yussuf Hassan Isak - Director General

   Signature........[signature]

   Date........1/11/2017

WASAARADDA QORSHEYNTA & ISKAASHIGA CAALAMIGA EE DOWLADDA KOONFUR GALBEED SOOMAALIYA
MINISTRY OF PLANNING & INTERNATIONAL COOPERATION
SOUTH WEST STATE OF SOMALIA
