# Supplementary material for: Cash-based assistance and the nutrition status of pregnant and lactating women in the Somalia food crisis: A comparison of two transfer modalities
Source: PLoS One. 2020 Apr 23;15(4):e0230989. doi: 10.1371/journal.pone.0230989 (PMC7179869; doi:10.1371/journal.pone.0230989)
Supplement: Supplementary file 2 [file pone.0230989.s001.docx]

Supplemental Table 1. Baseline characteristics by treatment group in unadjusted data and adjusted using stabilized inverse probability weighting*

	Unadjusted Data			Data Adjusted Using Stabilized Inverse Probability Weighting		
	Vouchers (N=166)	Mixed Transfers (N=288)	Standardized difference [1]	Vouchers (N=166)	Mixed Transfers (N=288)	Standardized difference [1]
Respondent Characteristics						
**Age**	28.6 ± 5.4	27.3 ± 5.1	**0.24**	27.4 ± 5.3	27.8 ± 5.4	0.07
**Education Level [2]**						
Never attended	81.9%	88.9%	**0.20**	86.4%	85.9%	0.02
Some primary but did not complete	13.3%	7.3%	**0.20**	9.4%	10.1%	0.02
Completed primary	4.8%	3.8%	0.05	4.1%	4.0%	0.01
**Marital Status**						
Married monogamous	62.7%	60.8%	0.04	62.6%	60.6%	0.04
Married polygamous	33.7%	38.5%	**0.10**	35.6%	37.9%	0.05
Widowed, Divorced, or Separated	3.6%	0.7%	**0.20**	1.7%	1.4%	0.02
**Beneficiary Type**						
Pregnant	46.4%	59.4%	**0.26**	53.5%	53.5%	0.00
Lactating	50.6%	38.2%	**0.25**	44.0%	43.8%	0.00
Pregnant and Lactating	1.8%	2.1%	0.02	1.9%	2.0%	0.01
Household Characteristics						
**Female Headed Households**	4.8%	1.7%	**0.17**	2.6%	2.7%	0.00
**Household Size**	6.6 ± 2.7	6.3 ± 2.6	**0.13**	6.3 ± 2.7	6.5 ± 2.8	0.04
**Children < 5 years in HH**	1.3 ± 0.7	1.2 ± 0.7	**0.14**	1.3 ± 0.7	1.3 ± 0.7	0.03
**Household Hunger Scale [3]**	1.4 ± 1.2	1.1 ± 1.1	**0.26**	1.3 ± 1.0	1.3 ± 1.2	0.02
**Meals consumed on preceding day**	2.6 ± 0.6	2.7 ± 0.6	**0.22**	2.8 ± 0.7	2.7 ± 0.6	**0.11**
Receipt of Food Assistance						
**Additional individual assistance received**	62.7%	85.4%	**0.54**	78.1%	76.8%	0.03

*Continuous variables presented as mean ± standard deviation; dichotomous and categorical variables presented as percentage

[1]Standardized differences less than 0.1 presumably indicate a “negligible” difference between groups; non-negligible differences are indicated in bold text

[2]No women completed secondary schooling

[3]Household Hunger Scale is six point scale of hunger in the past month where 0-1 is classified as little/no hunger, 2-3 as moderate hunger, and 4-6 as severe hunger
